# Visual Cu^2+^ Detection of Gold-Nanoparticle Probes and its Employment for Cu^2+^ Tracing in Circuit System

**DOI:** 10.1186/s11671-022-03742-z

**Published:** 2022-10-31

**Authors:** Tzu-Yu Ou, Chien-Feng Lo, Kuan-Yi Kuo, Yu-Pin Lin, Sung-Yu Chen, Chia-Yun Chen

**Affiliations:** 1 Department of Materials Science and Engineering, National Cheng Kung University, Tainan, 70101 Taiwan; 2 Green Energy and Environment Research Laboratories, Industrial Technology Research Institute, Tainan, 711010 Taiwan; 3 Hierarchical Green-Energy Materials (Hi-GEM) Research Centre, National Cheng Kung University, Tainan, 70101 Taiwan

**Keywords:** Optical sensing, Gold nanoparticle, Modification, Sensors

## Abstract

Highly sensitive, simple and reliable colorimetric probe for Cu^2+^-ion detection was visualized with the L-cysteine functionalized gold nanoparticle (LS-AuNP) probes. The pronounced sensing of Cu^2+^ with high selectivity was rapidly featured with obvious colour change that enabled to visually sense Cu^2+^ ions by naked eyes. By employing systemic investigations on crystallinities, elemental compositions, microstructures, surface features, light absorbance, zeta potentials and chemical states of LS-AuNP probes, the oxidation-triggered aggregation effect of LS-AuNP probes was envisioned. The results indicated that the mediation of Cu^2+^ oxidation coordinately caused the formation of disulfide cystine, rendering the removal of thiol group at AuNPs surfaces. These features reflected the visual colour change for the employment of tracing Cu^2+^ ions in a quantitative way.

**Supplementary Information:**

The online version contains supplementary material available at 10.1186/s11671-022-03742-z.

## Introduction

Copper ions (Cu^2+^) have been regarded as an essential constitute for simulating the biological functions, usually acting as a structural component of various enzymes and several proteins needed in metabolic processes [1–3]. Nevertheless, the excess accumulation of Cu^2+^ ions in human body might result in severe neurodegenerative and prion diseases. Herein, the urgent demand correlated with developing the detection technique that enabled to sensitively and selectively monitor Cu^2+^ ions. Extensive efforts have been made to address these challenges. For instance, dansyl-functionalized fluorescent film sensor was demonstrated that allowed to selectively sense Hg^2+^ and Cu^2+^ with sound sensitivity [4]. Besides, the detection of aqueous Cu^2+^ ions was presented through coumarin-salicylidene-based AIEgen [5]. While the associated detection sensitivities were quite promising, the employment of fluorescence spectroscopy was required to examine the emission effect. In addition, the real-time evaluation of Cu^2+^ ions was reported via the design of field effect transistor [6]. Although the correlated detection capability was reliable, the lack of sensing selectivity remained as unsolved issue against practical aspect. Recently, the doped perovskite quantum dots were employed as sensitive fluorescent probe, while the UV lights were required that stemmed against the employment on instant utilization [7]. Besides, it remained questionable whether the reported methods were practical on-site detection while freeing from the requirement of sophisticated instrumentation. Alternatively, the visual detection of Cu^2+^ ions [8–10] or other correlated metal ions [11–15] could be realized via a colorimetric route based on the effect of localized surface plasmon resonance (LSPR) [16–18], yet the underlying detection mechanism on the chemical-state examinations responding to the colour variation from selective detection of Cu^2+^ ions was rarely investigated. More importantly, it remained to be quite desirable for the practical employment of colorimetric sending of Cu^2+^ ions in circuit system with high detection selectivity and reliability. Herein, in this study, we presented a simple and selective colorimetric detection of Cu^2+^ ions by the naked eye through the L-cysteine modification of Au nanoparticles (LS-AuNPs) functioning as colour indicators. The underlying detection mechanism of colour transition was systematically revealed, where the practical employment of tracing Cu^2+^ ions in essential circuit components was performed.

## Experimental Section

### Materials

Trisodium citrate (Na_3_C_6_H_5_O_7_), L-cysteine (C_3_H_7_NO_2_S) and tetra-chloroauric (III) trihydrate (HAuCl_4_⋅3H_2_O) with purity of 99% were purchased from Sigma-Aldrich.

### Synthesis of Functionalized Au Nanoparticles

Preparation of LS-AuNPs was performed by mixing 0.5 ml aqueous HAuCl_4_ solution (0.02 M) with 0.03 g of citric acid under a magnetic stir for 30 min at 100 °C [19–22]. The obtained bare AuNPs (1.8 ml) were functionalized by mixing 5×10^-3^ M of L-cysteine (1 ml) with 0.2 ml deionized water and then incubated at room temperature for 2 h.

### Characterizations

Morphological and compositional analysis of obtained samples was characterized with scanning electron microscope (SEM, HITACHI SU6000) and energy-dispersive spectrometer (EDS, Oxford INCA), respectively. Microstructures of as-synthesized AuNPs were characterized by field emission transmission electron microscope (FE-TEM, JEOL JEM-2100F) with an acceleration voltage of 200 kV and a maximum line resolution of 0.14 nm, where samples were placed on carbon-coated copper grids and then dried prior to examination. X-ray diffraction (XRD) was performed to characterize the crystallinity of samples using a Cu Kα (*λ* = 0.15405 nm) as the radiation source at 30 kV with a scanning range of 30–55°. Surface functional groups of samples were analysed via Fourier Transform Infrared (FT-IR, JASCO/FT/IR 4600) spectrometer. Light-absorption spectra were measured with UV/Vis absorption spectrometer (Hitachi, U3900H) in the spectral range of 300–800 nm. Dynamic light scattering (DLS) was used to evaluate the size distribution of LS-AuNPs. X-ray photoelectron spectroscopy (XPS, PHI 5000 Versa Probe) analysis was conducted to investigate the chemical states of sample surfaces.

### Colorimetric Evaluation of Metal Ions

The as-synthesized colloidal LS-AuNP solutions were exposed to various concentrations of Cu^2+^ ions (10–130 μM) with fixed volume of 1 ml at room temperature. Colorimetric change was monitored by visual observation and recorded by a camera of cell phone. The light-absorption measurements were carried out using a UV–Vis spectrophotometer in the wavelength range of 300–800 nm. In practical employment, four various samples from electronic circuit components, including enameled wires, a piece of circuit board, nickel plate and speaker cable were tested to explore the practical sensing capabilities of AuNP probes. It should be noted that further purification was not required to conduct colorimetric detection. Briefly, the tested solutions were prepared by soaking the samples in 1.5 ml of nitric acid mixed with 8.5 ml of deionized water in gentle magnetic stir for 1 h. Subsequently, the pH values of as-prepared solutions were adjusted to close to 7 by adding few amounts of potassium hydroxide, and then filtered using a standard filter paper (45 µm). In the colorimetric detection, a drop of LS-AuNP probes was utilized that enabled to sensitively monitoring the Cu^2+^ ions of practical samples (1 ml of tested solutions), where the obvious colour change arising from the detection of Cu^2+^ ions could be less than 1 min.

## Results and Discussion

**Fig. 1:**
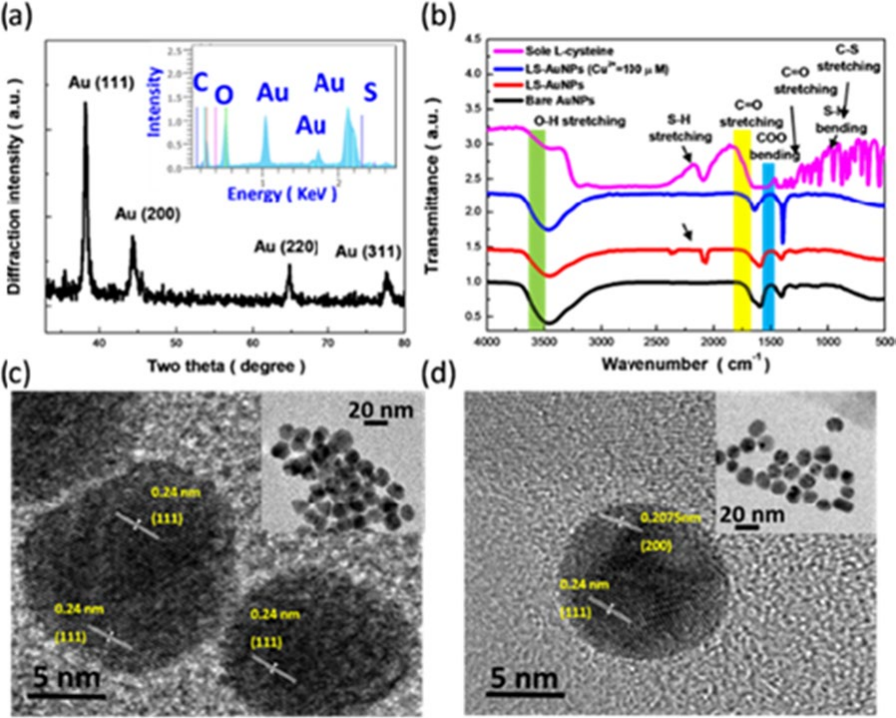
**a** XRD result of LS-AuNPs. Correlated EDS result was shown in the inset of Fig. 1a. **b** FTIR result and TEM images of LS-AuNP probes **c** before and **d** after sensing Cu^2+^ ions (50 μM)

Figure 1a presents the XRD characterizations of synthesized LS-AuNPs, indicating the formation of FCC crystallinity with discrete diffraction peaks corresponding to (111), (200), (220), and (311) planes. The correlated elemental compositions were examined with EDS analysis, where the obvious Au signals could be identified. Aside from that, one could explicitly find other compositional features including C, O and S signals, which suggested the successful surface modification of AuNPs with LS molecules. In addition, the spatial uniformity of LS-AuNP surfaces were examined with EDS mapping, as presented in Additional file 1. Surface features of obtained samples were characterized with FTIR analysis, where the corresponding results of bulk L-cysteine, bare AuNPs, LS-AuNPs, and LS-AuNPs with 50 μM of Cu^2+^ ions were displayed in the inset of Fig. 1a. In the spectrum of bulk L-cysteine, the transmission dips observed at 943 cm^-1^ and 2090 cm^-1^ were assigned to S–H bending and S–H stretching modes, respectively. In addition, C–S stretching, COO– bending, C=O stretching and OH– stretching vibration modes could be observed at 600–800 cm^-1^, 1200–1250 cm^-1^, 1500–1650 cm^-1^ and 3402 cm^-1^, respectively [23, 24]. By comparison, similar S–H bending mode could be also found in LS-AuNPs, verifying the effective functionalization of LS on AuNP surfaces. Nevertheless, the correlated S–H bending mode could not be found in either bare AuNPs or LS-AuNPs with Cu^2+^ ions, which implied the dramatic alteration of colloidal LS-AuNP features due to the removal of surficial LS molecules induced by Cu^2+^ ions. Other than that, the consistent transmission dips originated from COO– bending, C=O stretching and OH– stretching could be found in three samples including bare AuNPs, LS-AuNPs and LS-AuNPs with Cu^2+^ ions, which indicated that the core AuNPs maintained high structural stability after experiencing L-cysteine modification and succeeding Cu^2+^ detection. To shed light on the morphological transition of LS-AuNPs with Cu^2+^ ions (50 μM), TEM investigations were performed, as shown in the inset of Fig. 1c and d. The distinct colloidal morphologies were found from aggregated phenomena (without adding Cu^2+^, inset of Fig. 1c) toward fully dispersed state (with adding Cu^2+^, inset of Fig. 1d), whereas the microstructural crystallinity of AuNPs was maintained with the clear observations of (111) and (200) FCC lattices corresponding to XRD results. Thus, it could be speculated that the surface features of LS-AuNPs were modified with the addition of Cu^2+^ ions, and in turn remedied the morphological transition.

**Fig. 2:**
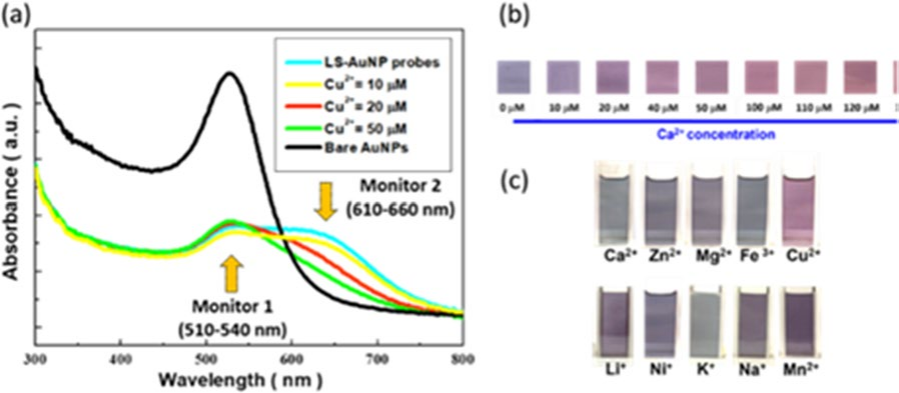
**a** Light-absorption spectra of various LS-AuNP probes. Colorimetric examinations for **b** detecting Cu^2+^ ions and **c** various metal ions with fixed 50 μM in concentration

The correlated light-absorption spectra of LS-AuNPs under the variations of Cu^2+^ concentrations along were examined, as shown in Fig. 2a. In addition, the light-absorption spectrum of bare AuNPs under dispersed state without experiencing LS modification was also presented. The main strong absorption peak located at 525 nm existed in bare AuNPs, originating from the LSPR effect that confined the incoming fields localized in the vicinity of AuNP surfaces [25–28]. The obvious red shift of such LSPR peak toward 610–660 nm was found in LS-AuNPs, where these features were driven by the intended aggregation of LS-AuNPs that contributed to the red-shift of spectral behaviours. When adding Cu^2+^ ions, the dramatic reduction of long-wavelength LSPR peak was encountered in line with the increase of Cu^2+^ ions, whereas in viewing of monitor 1 one could observe the light absorbance was increased with the increase of Cu^2+^ concentration. These implied that the LS-AuNPs gradually returned to dispersed phenomena while the Cu^2+^ ions were introduced. To visually examine the colour variations, the colorimetric table of LS-AuNP probes was presented under the wide variations of Cu^2+^ concentrations from 0 to 130 μM, as shown in Fig. 2b. In addition, the comparable colorimetric tables of suspensions including sole Cu^2+^ ions and bare AuNPs without LS modification were further presented in Additional file 1, which maintained to be opaque and red in colour regardless of Cu^2+^-ion concentrations, respectively. Evidently, the pronounced colour change visually by naked eyes for monitoring Cu^2+^ analytes was demonstrated, as evidenced in Fig. 2b. The results indicated that the main trend toward colour change was from dark purple toward to wine red, indicating the morphological transition of LS-AuNPs from aggregated state toward dispersed condition, respectively, where the visual results were in good agreement with the spectral findings (Fig. 2a). It should be noted that the distinct surface functionalization of AuNP probes led to different morphological change when sensing Cu^2+^ ions. For instance, Wang reported the surface modification of AuNPs with multiple antibiotic resistance regulator as the biorecognition elements that displayed the opposite colour change from red to purple when sensing Cu^2+^ ions compared with this work [29]. Aside from that, the designed LS-AuNP probes further enabled to the employment for quantitatively detecting Cu^2+^ ions, as detailed in Additional file 1. This was accomplished by monitoring the light-absorption ratio of 620/525 nm under the variations of Cu^2+^-ion concentrations. The spectral employments rendered the sound quantitative fitting of light absorptance in terms of A620/A520 nm with respect to the concentration of Cu^2+^ ions, with the high *R*^2^ value of 0.96, which indicated the change of LS-AuNP colour could readily correlate with the practical Cu^2+^ concentration. In addition, LOD and LOQ results were also presented in Additional file 1.

Another crucial aspect correlated with the detection selectivity was explored in Fig. 2c. By testing the present colour of LS-AuNPs with various metal ions (50 μM), only the case of detecting Cu^2+^ could result in the substantial colour variation toward the distinct wine red driven by the effective aggregated/dispersed transition of LS-AuNP colloids, and essentially provided the colorimetric monitoring of Cu^2+^ ions without the assistance of any sophisticated instrument. Nevertheless, the morphological transition could not be initiated in the presence of other metal ions as the displayed colours maintained to be dark purple or grey with trivial colour change. However, it should be also pointed out that the designed LS-AuNP probes showed the poor detection selectivity on sensing low concentration of Cu^2+^ ions (< 10 µm) in the co-existence of Pb^2+^ or Cd^2+^ ions, as demonstrated in Additional file 1.

**Fig. 3:**
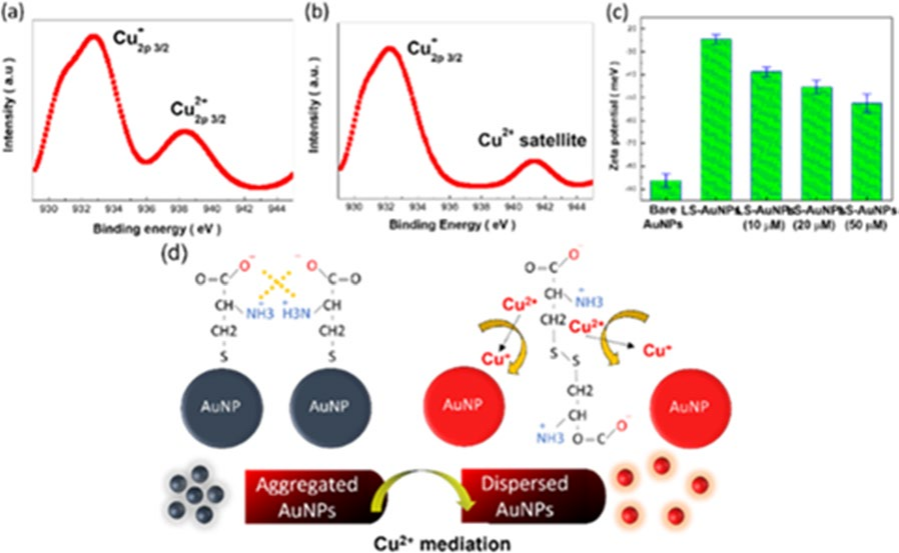
XPS results of LS-AuNP probes for detecting Cu^2+^ ions: **a** 20 μM and **b** 50 μM. **c** Zeta potentials of different LS-AuNP samples. **d** Schematic illustration for detecting Cu^2+^ ions

In colorimetric detection strategy of AuNP-based indicators, the colour change might strongly correlate with the surface configurations [30–32]. Figure 3a and b demonstrates the chemical states of LS-AuNP probes in the presence of 20 μM and 50 μM of Cu^2+^ ions, respectively. At 20 μM of Cu^2+^ concentration, two characteristic photoelectron peaks corresponding to Cu^+^_2p3/2_ (932.75 eV) and Cu^2+^_2p3/2_ (938.36 eV) were envisioned, indicating the introduced Cu^2+^ ions were partially reduced to Cu^+^ ions while interacting with LS-AuNP probes. Such spectral circumstances were altered while 50 μM of Cu^2+^ ions were added, where the similar Cu^+^_2p3/2_ (932.73 eV) along with relatively weak Cu^2+^ satellite peak (941.51 eV) could be observed, as shown in Fig. 3b [33, 34]. It could be speculated that the majority of added Cu^2+^ ions were oxidized to Cu^+^ ions, and in turn altered the LS ligands on LS-AuNP surfaces. Another evident examination was performed by zeta-potential measurements, as shown in Fig. 3c. By comparing the high average zeta potential of LS-AuNPs (− 24.5 meV), the gradual reduction of zeta potential was found eventually down to − 52.5 meV from LS-AuNPs with 50 μM Cu^2+^ ions. These features could be interpreted by the fact that the negative polarity of LS-AuNPs was gradually decreased via the mediation of Cu^2+^ oxidation, in turn losing their dispersed stability. Thus, the Cu^2+^-mediated LS-AuNPs favourably aggregated with neighbouring counterparts rather than maintaining the inherently dispersed features, which resulted in the visual colour variations. Taking together, the detection mechanism could be conceptually elucidated, as illustrated in Fig. 3c. Through LS functionalization, thiol groups were formed that facilitated the coordination of LS with AuNP surfaces. It should be noted that the self-aggregation of LS-AuNPs was energetically favourable because the involvement of dipole–dipole coupling between neighbouring LS-AuNPs, which reflected the dark purple visually observed by the naked eye. With introducing Cu^2+^ analytes, oxidation of Cu^2+^ drove the following reaction,

1 $${\text{L}} - {\text{cysteine}} + {\text{Cu}}^{2 + } \to {\text{Cu}}^{ + } + 1/2\;{\text{disulphide}}\;{\text{cystine}}$$

Accordingly, the mediation of Cu^2+^ oxidation coordinately caused the formation of disulphide cystine, rendering the removal of thiol group at AuNPs surfaces. As a consequence, instant variation of indicator colour toward red due to dispersed nature was encountered, which could be effectively implemented for practical colorimetric detection of Cu^2+^ ions.

**Fig. 4:**
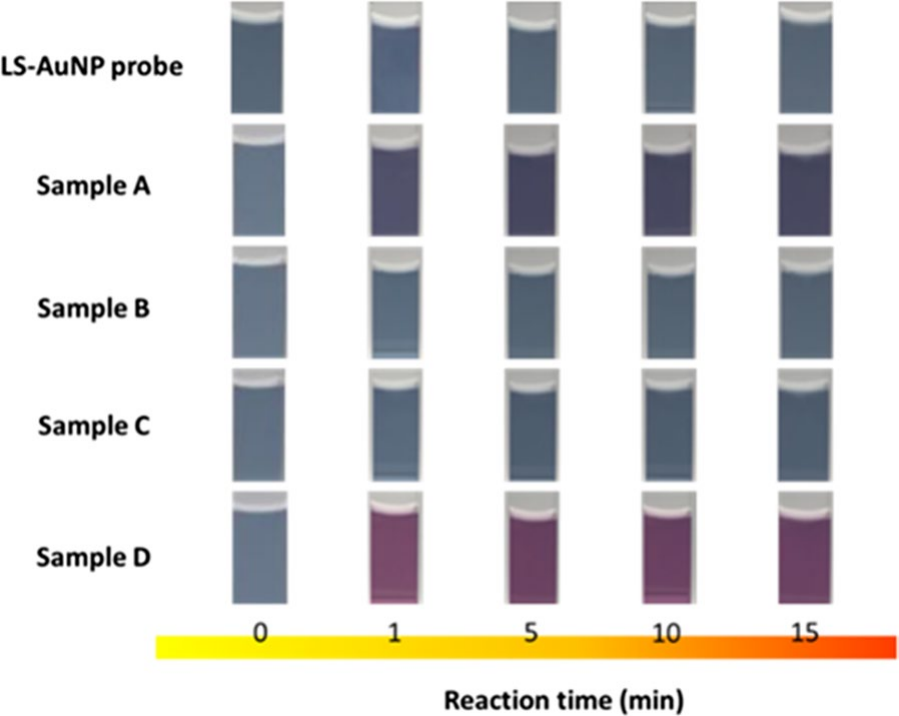
Visual colorimetric table of four different samples with respect to reaction time

**Fig. 5:**
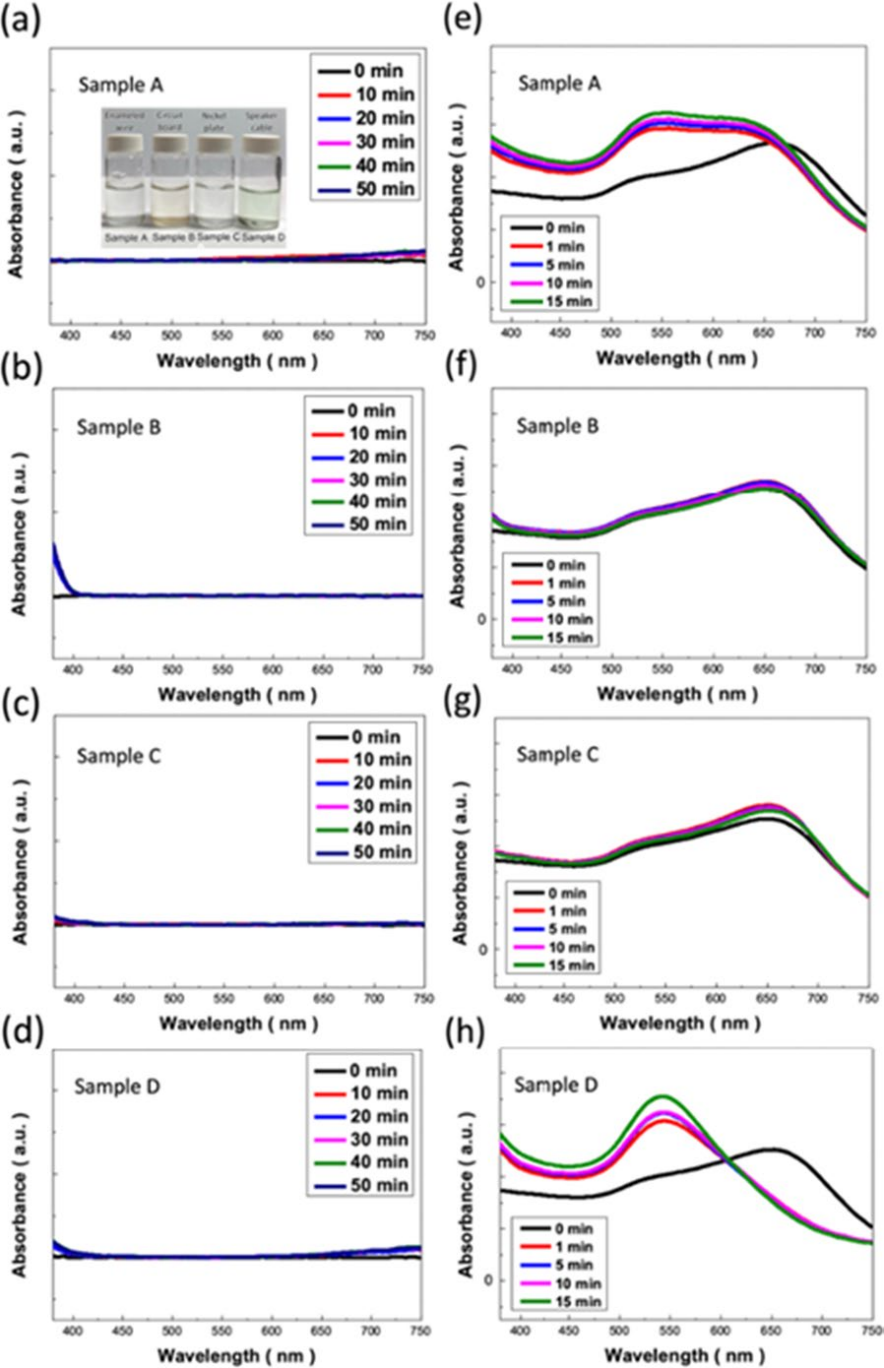
UV/Vis absorption spectra of bare tested solutions: **a** Sample A **b** Sample B **c** Sample C and **d** Sample D, respectively. The inset figure of Fig. 5a presented the photographs of tested solutions for further detection. UV/Vis absorption spectra of **e** Sample A **f** Sample B **g** Sample C and **h** Sample D in the presence of LS-AuNP probes, respectively, where the obvious colorimetric detection for selectively sensing Cu^2+^ ions could be evidenced

Finally, the practical employment for Cu^2+^ tracing in conventional circuit system was explored. Four essential samples from electronic circuit components, including enameled wires (Sample A), a piece of circuit board (Sample B), nickel plate (Sample C) and speaker cable (Sample D) were tested to explore the practical sensing capabilities of LS-AuNP probes, as detailed in Table 1. The correlated colorimetric comparisons with respect to the detection durations were presented in Fig. 4, where two tested samples without Cu content, including Sample B and Sample C, remained the consistent colour of original LS-AuNP indicators regardless of detection time. By contrast, the clear colour change of both Sample A and Sample D possessing Cu contents could be visually observed by naked eyes, where the detection time required for visual observation could be less than 1 min, showing the effective and rapid detection performances. Moreover, one could not find further variation of demonstrated colour in tested solution at least 30 min of detection time, indicating the stable and reliable indication of Cu^2+^ sensing. To further get insight into the sensing capabilities of developed LS-AuNP probes, the comparisons of UV/Vis absorption spectra obtained from bare tested solutions and addition of LS-AuNP indicators in tested solutions were displayed, as shown in Fig. 5. For the spectral measurement of bare tested solutions, no clear absorption characteristics could be found from entire measured spectra of all four tested samples, as shown in Fig. 5a–d, respectively. This could be attributed to the very low concentrations of metal ions that were incapable of being detected by the spectrometer. Nevertheless, with the addition of LS-AuNP indicators in tested solutions, the clear variation of absorption spectra could be found in Sample A (Fig. 5e) and Sample D (Fig. 5h), whereas the measured spectra remained to be consistent in Sample B (Fig. 5f) and Sample C (Fig. 5g), visualizing the high detection selectivity toward sensing Cu^2+^ ions via LS-AuNP probes in practical assessment.

**Table 1:** Detailed information and correlated sensing capabilities of four practical samples

Sample	Electronic materials (product name)	Company	Components (weight %)	Colorimetric sensing	Spectral measurement	Repeatability
A	Enameled wire (UEW)	Jung Shing wire	Copper (98%) PU (2%)	**√** (Sensitive)	Not detectable	√
B	Circuit board (EIC-16010)	E-call enterprise Co., Ltd	ABS plastic (29%) POM plastic (45%) Carbon steel (1%) Phosphor bronze (17%) Nickel (8%)	X	Not detectable	N/A
C	Nickel plate (N6)	Centenary materials Co., Ltd	Nickel (99.5%)	X	Not detectable	N/A
D	Speaker cable (SPK-D-105A)	DC cable	Copper (10%) Silver (24%) PVC (66%)	√√ (Highly sensitive)	Not detectable	√

## Conclusions

We presented a highly selective and visual Cu^2+^ detection technique via facile functionalization of AuNPs with LS molecules. Detailed crystallinities, chemical compositions, surface features, microstructures, zeta potentials and chemical states were investigated to unveiled the underlying detection mechanism for visually sensing the wide range of Cu^2+^-ion concentrations, where the LS-AuNP-based colorimetric indicators achieved the detection minimum down to 10 μM. Through the in-depth elucidation of sensing mechanism, we anticipated that these visual designs could practically monitor the trace Cu^2+^-based pollutants and extend to the employment of pollutant treatment.

## Supplementary Information

**Additional file 1: Figure S1.** Characterizations: EDS mapping results, diffraction patterns, size distributions of LS-AuNPs. **Figure S2.** Relative colorimetric examinations. **Figure S3.** Quantitative detection of Cu^2+^ ions. **Figure S4.** Repeatability examinations of LS-AuNP probes for monitoring Cu content of a piece of speaker cable.  **Figure S5.**  Detection of mixed metal ions (Cu^2+^, Pb^2+^, Cd^2+^).

## Data Availability

The datasets supporting the conclusions of this article are included within the article.

## Abbreviations

XRD: X-ray diffraction
EDS: Energy-dispersive spectrometer
FTIR: Fourier transform infrared spectrometer
DLS: Dynamic light scattering
XPS: X-ray photoelectron spectrometer
TEM: Transmission electron microscope
SEM: Scanning electron microscope
