# Supplementary material for: Visual Cu2+ Detection of Gold-Nanoparticle Probes and its Employment for Cu2+ Tracing in Circuit System
Source: Nanoscale Res Lett. 2022 Oct 31;17:104. doi: 10.1186/s11671-022-03742-z (PMC9622959; doi:10.1186/s11671-022-03742-z)
Supplement: Supplementary file 1 — Additional file 1: Figure S1. Characterizations: EDS mapping results, diffraction patterns, size distributions of LS-AuNPs. Figure S2. Relative colorimetric examinations. Figure S3. Quantitative detection of Cu2+ ions. Figure S4. Repeatability examinations of LS-AuNP probes for monitoring Cu content of a piece of speaker cable. Figure S5. Detection of mixed metal ions (Cu2+, Pb2+, Cd2+). [file 11671_2022_3742_MOESM1_ESM.docx]

**Supplemental files**

**Visual Cu^2+^ detection of gold-nanoparticle probes and its employment for Cu^2+^ tracing in circuit system**

Tzu-Yu Ou^1^, Chien-Feng Lo^1^, Kuan-Yi Kuo^1^, Yu-Pin Lin^2^, Sung-Yu Chen^2^ and Chia-Yun Chen^1,3*^

^1^Department of Materials Science and Engineering, National Cheng Kung University, Tainan 70101, Taiwan

^2^Green Energy and Environment Research Laboratories, Industrial Technology Research Institute, Tainan 711010, Taiwan

^3^Hierarchical Green-Energy Materials (Hi-GEM) Research Centre, National Cheng Kung University, Tainan 70101, Taiwan

*Correspondence: timcychen@mail.ncku.edu.tw

**S1 Characterizations: EDS mapping results, diffraction patterns, size distributions of LS-AuNPs**


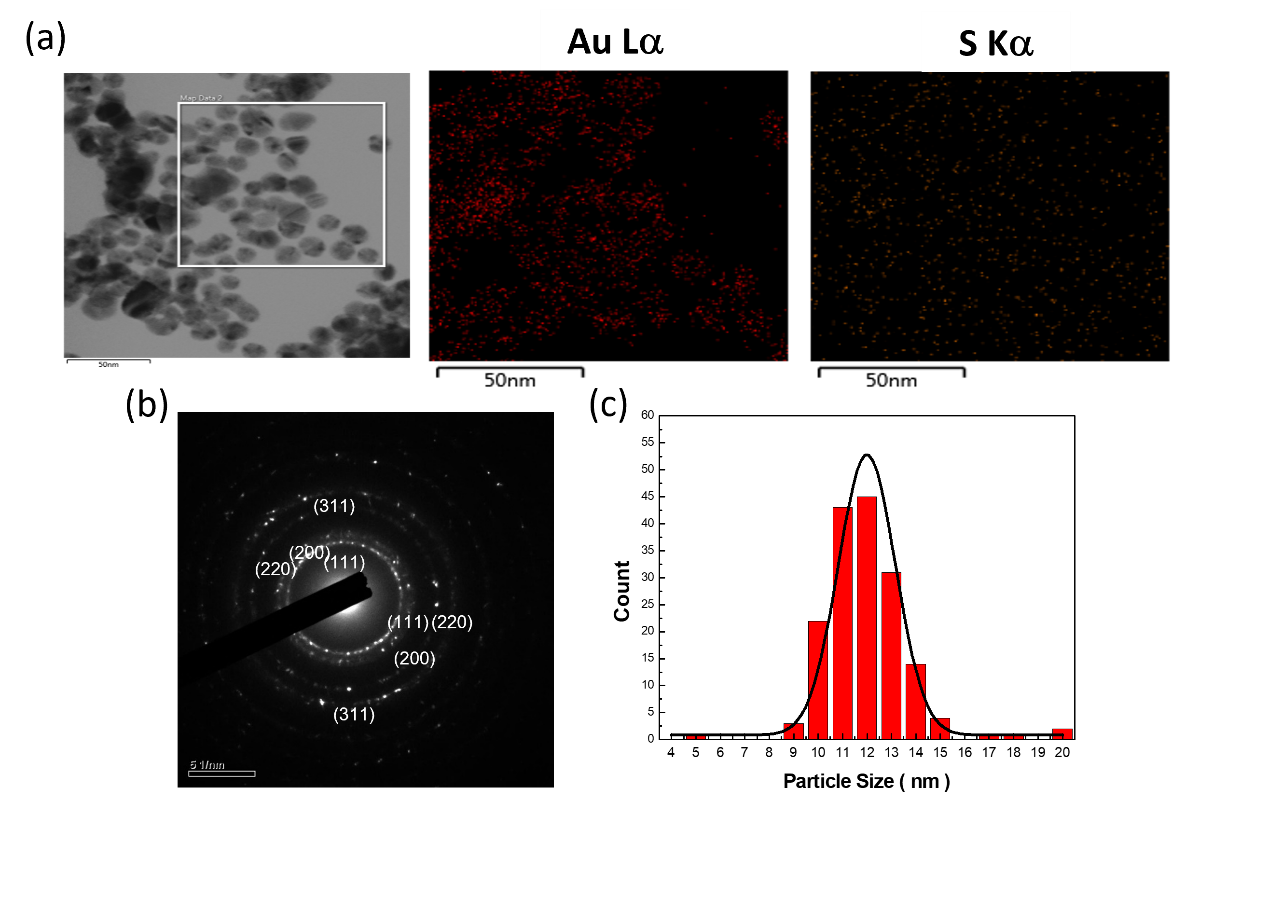


Fig. S1 (a) EDS mapping of formed LS-AuNPs. The elemental distributions of Au and S signals evidenced the uniform formation of LS-decorated AuNPs. (b) Diffraction patterns of LS-AuNPs. The findings corresponded well with the HRTEM and XRD results. (c) Size distribution of LS-AuNPs. The average dimension was found to be 12.4 nm.

**S2 Relative colorimetric examinations**


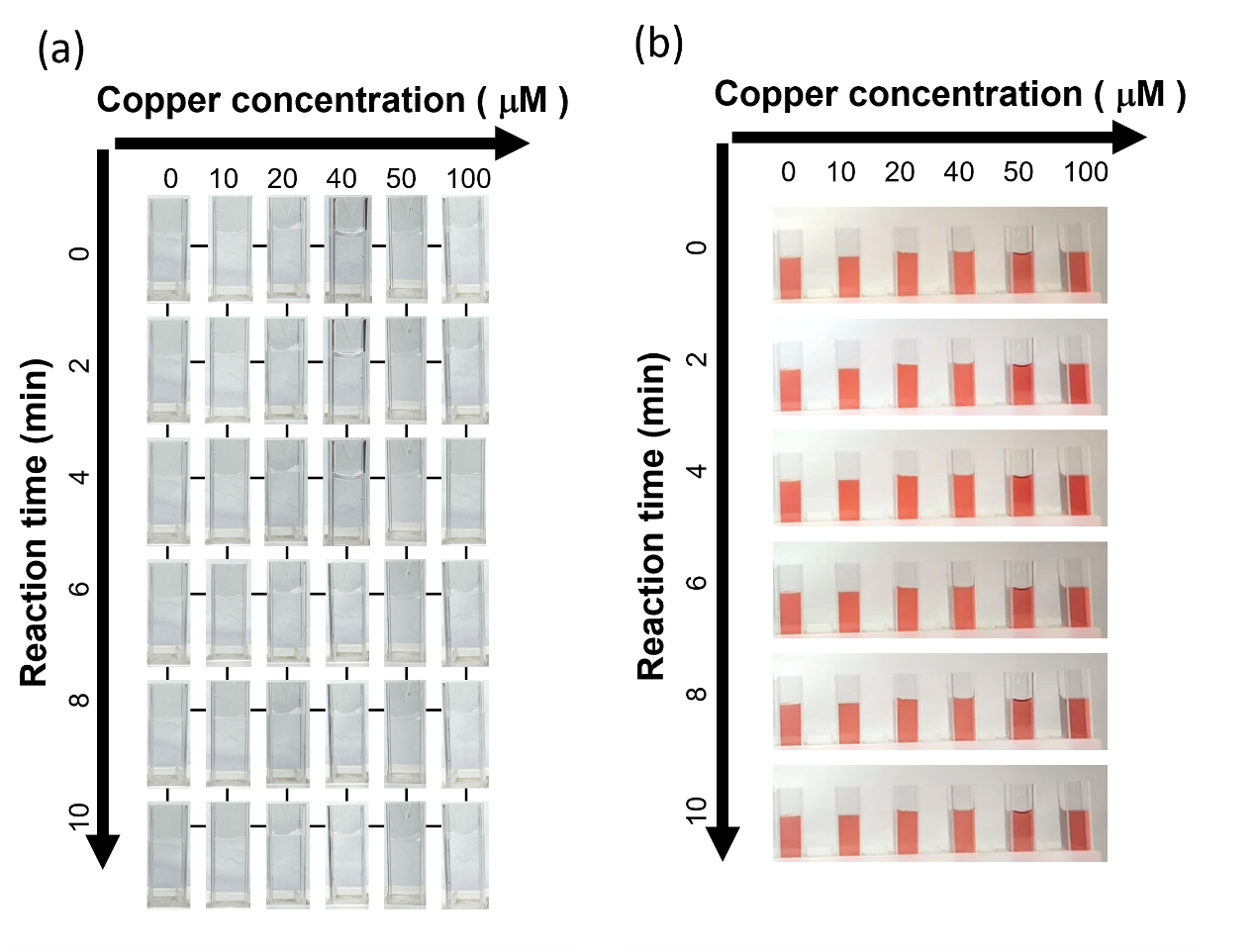


Fig. S2 Colorimetric tables of (a) sole Cu^2+^ ions and (b) bare AuNPs without LS modification. The results indicated that the sole Cu^2+^-ion solutions were transparent regardless of the tested Cu^2+^ concentrations or reaction durations. In addition, without LS modification, the color of AuNPs stayed to be wine red regardless of the tested Cu^2+^ concentrations or reaction durations.

**S3 Quantitative detection of Cu^2+^ ions**


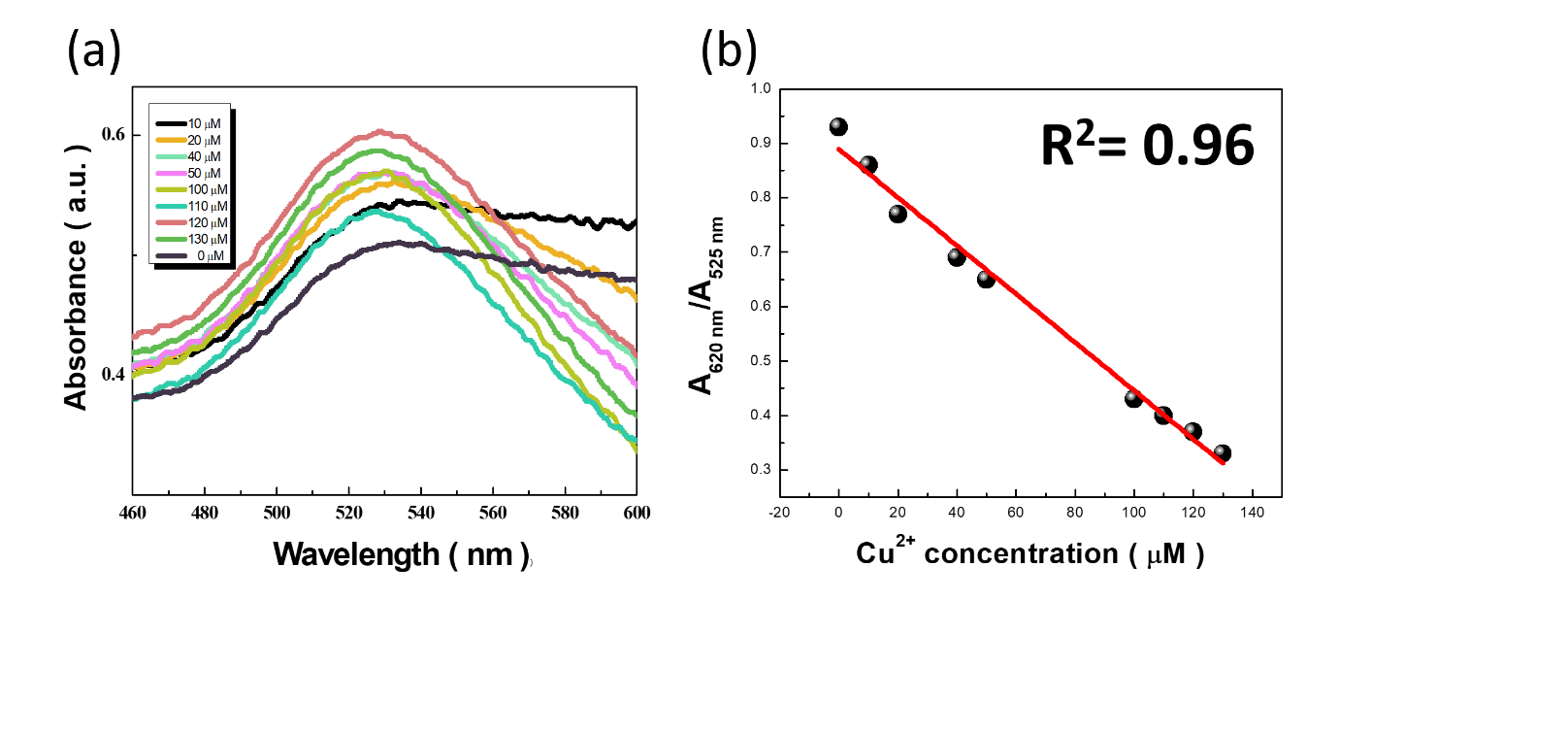


Fig. S3 (a) Light-absorption spectra of LS-AuNPs in the presence of various Cu^2+^-ions concentrations. (b) Monitoring the light-absorption ratio of 620 nm/525 nm under the variations of Cu^2+^-ion concentrations. These spectral employments rendered the sound quantitative fitting of light absorptance in terms of A_620 nm_/A_520 nm_ with respect to the concentration of Cu^2+^ ions, with the high R^2^ value of 0.96, which indicated the change of LS-AuNP color could readily relate to the practical Cu^2+^ concentration.

Fig. S4 Evaluations of LOD and LOQ values of LS-AuNP probes for the colorimetric detection of Cu^2+^ ions.

Based on the measured results from Fig. S4, the value of standard deviation was calculated to be 0.153, and both limit of detection (LOD) and limit of quantification (LOQ) were quantitatively evaluated, where the LOD and LOQ were found to be 35.06 μM and 106.25 μM, respectively. These results were comparable with previous literature [S1-S3], while the present work showed the sound detection selectivity and has been successfully employed for Cu^2+^ tracing in circuit system. It should be also noted that the LOD of LS-AuNP probes was lower than the literature [S4], whereas the UV/Vis spectrometer was required for spectral sensing in Ref. S4.

1. Liu L, Xie M-R, Fang F, Wu Z-Y (2018) Microchemical Journal 139:357-62.
2. Hu C Y, Jiang Z W, Huang C Z, Li Y F (2021) Microchimica Acta 188:272.
3. Aydin Z, Keles M (2020) ChemistrySelect 5(25):7375-81.
4. Park S M, Saini S, Park J E, Singh N, Jang D O (2021) Tetrahedron Letters 73:153115.

S4 Repeatability examinations of LS-AuNP probes for monitoring Cu content of a piece of speaker cable.


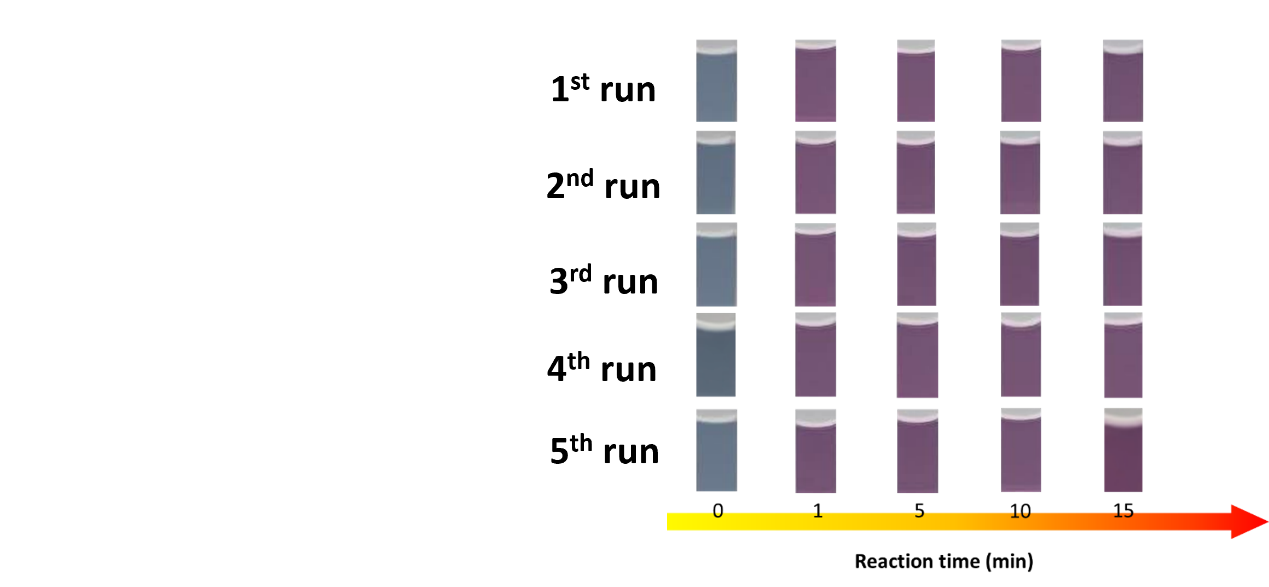


Fig. S5 LS-AuNP repeatability test for examining Cu content of a piece of speaker cable. All the results remain approximately similar, evidencing the detection reliability of the LS-AuNP probes.

**S5 Detection of mixed metal ions (Cu^2+^, Pb^2+^, Cd^2+^)**

**
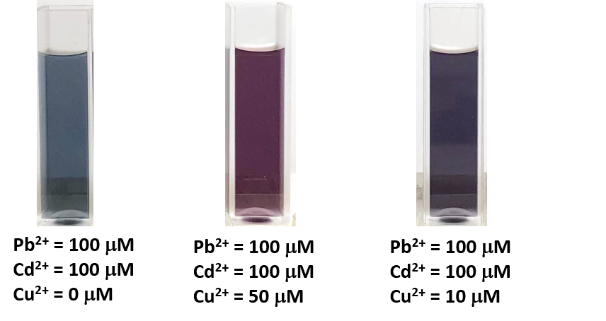
**

Fig. S6 Detection of mixed metal ions using LS-AuNP probes. One could find the clear color change from purple to red when adding Cu^2+^ ions, whereas the co-existence of Pb^2+^ and Cd^2+^ ions did not strongly disturb the detection of Cu^2+^ ions. It should be pointed out that the detection seemed to be slightly influenced when the concentration of Cu^2+^ was lower than 10 μM under the co-existence of Pb^2+^ or Cd^2+^ ions.
